# Aerial Track‐Guided Autonomous Soft Ring Robot

**DOI:** 10.1002/advs.202503288

**Published:** 2025-04-25

**Authors:** Fangjie Qi, Caizhi Zhou, Haitao Qing, Haoze Sun, Jie Yin

**Affiliations:** ^1^ Department of Mechanical and Aerospace Engineering North Carolina State University Raleigh NC 27695 USA

**Keywords:** liquid crystal elastomers, soft machine

## Abstract

Navigating in three‐dimensional (3D) environments with precise motion control is challenging for soft robots due to their inherent flexibility. Inspired by aerial trams, here, an autonomous soft twisted ring robot is reported capable of navigating pre‐defined tracks in 3D space under constant photothermal actuation, without requiring spatiotemporal control of actuation sources. Made of liquid crystal elastomers, the ring robot, suspended on thread‐based tracks, self‐flips around its centerline when exposed to constant infrared light. Curling the twisted ring around tracks converts its self‐rotary motion into autonomous linear movement via screw theory. This mechanism enables the autonomous robot to adapt to tracks of various materials and micron‐to‐millimeter sizes, overcome obstacles like knots on tracks, transport loads over 12 times its weight, ascend and descend steep slopes up to 80°, and navigate complex paths, including circular, polygonal, and 3D spiral tracks, as well as loose threads with dynamically changing shapes.

## Graphical abstract

Controlling motion paths in soft active material‐based robots is challenging due to their inherently high degrees of freedom. Inspired by aerial trams, a new class of autonomous, track‐guided soft robots composed of twisted liquid crystal elastomer rings are reported. Under constant infrared light, these robots can self‐navigate along predefined 3D tracks without spatiotemporal control of light sources.

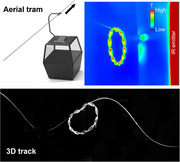

## 1 Introduction

**Figure 1:**
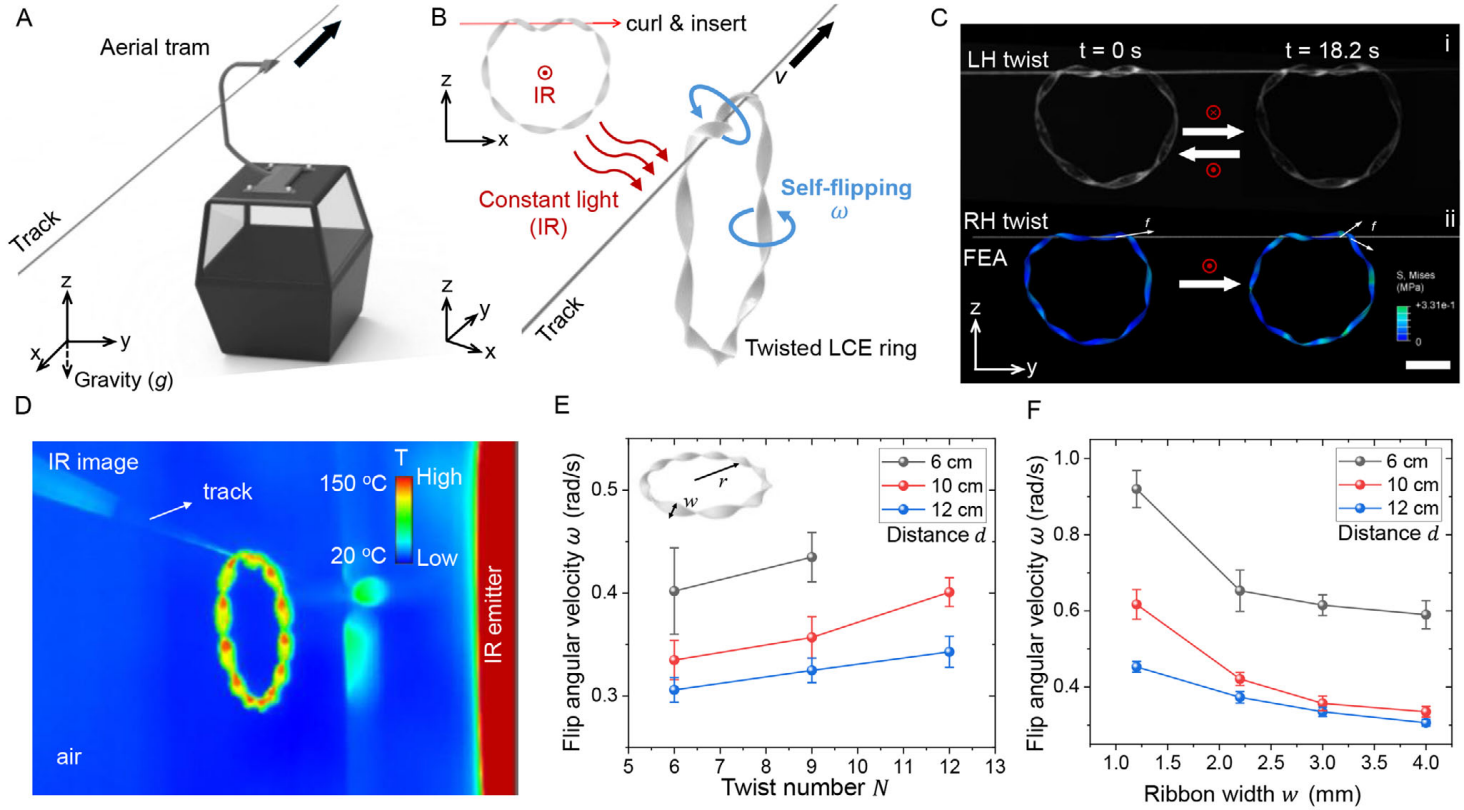
Aerial tram‐like autonomous soft twisted liquid crystal elastomer (LCE) ring robot under constant photothermal actuation. A) Schematic of an aerial tram. B) Schematic of an autonomous soft twisted LCE ring robot that is curled around a track to convert self‐flipping motion into linear movement along the track when exposed to constant infrared (IR) light perpendicular to the ring plane. The inset shows the hanging‐on‐track process by inserting the thread into a curling ring. C) Experimental (i) and finite element analysis (FEA) simulation (ii) results on twisted LCE rings moving along a thin fishing line track (diameter = 0.27 mm) under constant IR light (front views, *y*‐*z* plane). The moving directions can be tuned by reversing either the twisted chirality of left‐handed (LH) and right‐handed (RH) twists or incident light directions along the positive or negative *x*‐axis. Scale bar: 1 cm. D) IR image of the curled and twisted LCE ring on a fishing line track when exposed to an IR emitter. E) The self‐flipping angular velocity on a track *ω* as a function of twist number *N* at different distances from the IR emitter. The ribbon width *w* and ring radius *r* are kept as 3 and 22 mm, respectively. F) The self‐flipping angular velocity on a track *ω* as a function of ribbon width *w* at different distances from the IR emitter. The twist number *N* and the ring radius *r* are kept as 9 and 22 mm, respectively.

Inspired by the remarkable movements of plants and animals, bio‐inspired soft‐bodied robots aim to replicate the diverse motions observed in their natural counterparts,^[^ ^1^, ^2^, ^3^ ^]^ including crawling,^[^ ^4^ ^]^ running,^[^ ^5^ ^]^ jumping,^[^ ^6^ ^]^ swimming,^[^ ^7^ ^]^ and flying.^[^ ^8^ ^]^ Recent advances in soft mobile robots have expanded their motion capabilities from land^[^ ^4^ ^]^ to underwater environments,^[^ ^7^ ^]^ and into the air^[^ ^9^ ^]^ and inside the human body.^[^ ^10^, ^11^, ^12^ ^]^ Among these modes of movement, track‐guided motions stand out from free movements by relying on a physical track, a concept prevalent in both nature and engineering but still largely unexplored in soft robotics. For example, in nature, plant tendrils climb by curling around supports, using them as tracks for growth.^[^ ^13^ ^]^ In engineering, track‐guided transportation systems such as trolleys, railways, and aerial trams (**Figure** **1**A) have been essential means of transport. The aerial tram system conveys the cabin to certain locations by arranging the track paths and positions. Benefiting from predefined routes, track‐guided systems allow for precise yet simplified path control, as well as the adaptability to navigate challenging terrains by adjusting tracks.

In contrast, despite recent progress in soft mobile robots, achieving path control remains a challenge due to the continuum deformability and compliant nature of their soft bodies.^[^ ^14^ ^]^ The inherent flexibility and deformability allow for multiple, even infinite, degrees of freedom (DOF), enabling a wide range of configurations for enhanced adaptability.^[^ ^14^ ^]^ However, this flexibility complicates motion prediction, path planning, and control.^[^ ^14^, ^15^, ^16^ ^]^ Furthermore, the free movements of soft robots are highly susceptible to environmental interactions and external disturbances. For example, tethered tubes or wires can interfere with the motion driven by fluidic^[^ ^17^ ^]^ or electroactive actuations;^[^ ^18^ ^]^ the untethered motions are sensitive to perturbations in remote actuation by means of spatiotemporal control of external stimuli,^[^ ^19^, ^20^, ^21^ ^]^ such as light, photothermal, and magnetic fields.

Track‐guided motion provides a potentially promising way to achieve path control in soft mobile robots, since the geometric constraints of tracks could reduce the number of DOF in deformation and motion, and the soft body could allow adaptive interactions between soft robots and tracks to adapt to the track changes. However, it remains challenging to achieve track‐guided soft robots that can autonomously follow predefined tracks without the need for external spatiotemporal control of actuation sources. To tackle the challenge, recent studies show promising results by leveraging constant thermal or light fields to induce autonomous track‐free movements,^[^ ^22^, ^23^, ^24^, ^25^, ^26^, ^27^, ^28^, ^29^, ^30^, ^31^ ^]^ and track‐guided periodic orbiting^[^ ^32^ ^]^ or linear motions.^[^ ^33^ ^]^ For example, we report that, when placed on a hot surface with a constant temperature, a defected twisted liquid crystal elastomer (LCE) ring can periodically self‐orbit along enclosed boundary walls of various shapes,^[^ ^32^ ^]^ driven by self‐flipping induced alternating cooling and heating. Similarly, a pierced liquid crystal network strip hanging from a horizontal thread was demonstrated to autonomously move along the straight thread under a constant laser beam via light shadowing‐induced self‐oscillation.^[^ ^33^ ^]^ Notably, aerial tracks, which release the limitations of planar motion on ground,^[^ ^22^, ^23^, ^24^, ^28^, ^29^, ^30^, ^31^, ^34^, ^35^ ^]^ offer tremendous potential for navigation and transport in three‐dimensional (3D) space (Table S1, Supporting Information for a comparison of LCE‐based soft actuators). Despite these advances, the challenge of achieving autonomous spatial navigation via plane and space curves under constant thermal or light fields remains largely unexplored.^[^ ^32^, ^33^ ^]^

Here, we report an aerial tram‐like autonomous twisted LCE ring robot capable of navigating and transporting in 3D space under constant, remote photothermal actuation, without the need for external spatiotemporal control. By partially curling and hanging on a thread, the ring robot autonomously moves along the track when exposed to constant infrared (IR) light (Figure 1B). Similar to a linear screw mechanism, the ring robot utilizes the adaptive interaction between the twisted soft ring and the thread to convert self‐rotary motion into autonomous linear movement via screw theory. We also explore the ring bot's adaptability to tracks made from various materials, ranging from soft and flexible to hard, and in different sizes, from micron to millimeter scales. It also exhibits strong load‐carrying capabilities, autonomously transporting over 12 times its own weight. Lastly, we demonstrate its unique ability for track‐guided autonomous navigation in 3D space under constant light field, including self‐motion along the plane and space curves with steep slopes and varied geometries, as well as challenging tracks such as loose flexible threads and spiral threads with varying curvature and torsion.

## 2 Results

### 2.1 Autonomous Rotary to Linear Motion Conversion

Figure 1B schematically illustrates the design of the aerial tram‐like autonomous twisted LCE ring robot. The ring is fabricated by bending and bonding the two ends of a twisted LCE ribbon, synthesized by following the previously reported two‐stage reaction method^[^ ^32^, ^36^ ^]^ (Figures S1 and S2, Supporting Information; see Experimental Section for more details). The nematic‐isotropic transition temperature of the as‐synthesized LCE *T_NI_* is near 80 °C. When curled onto a thread (e.g., a thin fishing line) following its twisting chirality, the ring robot hangs on the track (Figure 1B,C). Exposure to constant IR light over the twisted ring plane (*y‐z* plane) from an IR emitter induces global self‐flipping rotation around its centerline. This self‐flipping is driven by a temperature gradient‐induced torque across the ribbon's width, where the side closer to the IR light heats up more, shrinking more than the opposite side, as shown in the IR image of Figures 1D and S2 (Supporting Information). This is similar to the self‐flipping of soft active rings on a hot plate.^[^ ^24^, ^31^, ^32^ ^]^ However, here the ring self‐flips while hanging in the air without support. Like a linear screw system, the local contact interaction between the rotating twisted ring and the straight thread converts its rotary motion into linear movement (Figure 1C; Movie S1, Supporting Information). Upon exposure to IR light, the ring initially undergoes accelerated motion, driven by the temperature gradient‐induced torque and friction forces. It then transits into a steady state. The critical condition for initiating the motion can be identified by measuring the peak acceleration (Figure S3, Supporting Information). Once the ring reaches steady–state motion, it moves along the track at a nearly constant linear velocity, with slight fluctuations due to passing through the binding point (Figure S3, Supporting Information). Although sliding friction is near zero, as indicated by the stable velocity range, two torques remain: one is the driving torque generated by the heating source, the other is the torque from rolling friction, which is effectively balanced by gravity (Figure S4, Supporting Information). Continuous, autonomous, meter‐range linear movement on a track can be achieved under constant photothermal actuation from an array of fixed IR emitters (Figure S5, Supporting Information). The ring's moving direction is controlled by its twisting chirality^[^ ^32^ ^]^ and the light's incident direction. With light incident along the positive *x*‐axis, a left‐handed (right‐handed) twisted ring self‐rotates clockwise (counterclockwise) around the thread, generating friction forces *f* that are opposite and tangential to the rotational direction of the contacted ring with the thread, as shown in the finite element analysis (FEA) simulation results in Figure 1C‐ii (see Experimental Section for more details on FEA simulation). For a right‐handed twisted ring, the resultant friction forces point to the positive *y*‐axis, driving the ring to move linearly to the right (Figure 1C‐ii; Movie S2, Supporting Information). The FEA simulation result is consistent with the experiments. Reversing the twisting handedness or light direction switches the movement direction (Figure 1C).

The autonomous rotary‐to‐linear motion conversion depends on several factors: the incident angle *β* relative to the ring plane, the light intensity determined by the distance *d* between the light source and the ring, the number of curls around the thread *n_c_*, and the geometry of the twisted ring. For optimal performance with a given ring geometry, the highest linear speed is achieved when i) *β* is close to 0, meaning the light is perpendicular to the ring plane (Figure S6, Supporting Information), ii) the ring is placed at an intermediate distance *d* from the light source (Figure S7, Supporting Information), and iii) *n_c_* equals 1, meaning it curls only once around the thread (Figure S8, Supporting Information). As shown in Figure S6 (Supporting Information), as *β* increases from −90° (parallel to the ring plane) to 90°, the ring remains stationary at *β* = ± 90°, with the peak speed of 2.2 mm s^−1^ occurring at *β* = 0°. The stationary ring at *β* = ± 90° results from the lack of a temperature gradient across the ribbon width when the light source is directly above (*β* = 90°) or beneath (*β* = −90°) the ring. Similarly, when only part of the ring is exposed to light, it remains stationary due to insufficient torque to rotate the entire ring from localized heating. Figure S7 (Supporting Information) shows that as *d* increases from 4 to 9 cm, the longest travel distance is achieved at an intermediate distance of *d* = 4 cm. When *d* is too small, the ring overheats due to the high‐temperature field, leading to stationary behavior. Figure S8 (Supporting Information) demonstrates that when the ring is simply hanging without curling, it exhibits chaotic oscillatory motion driven by its self‐rotation with low friction. This chaotic motion transforms into linear movement after adding one curl around the thread with more contacts, allowing for effective rotary‐to‐linear motion conversion. However, more than one curl induces higher pre‐stress and friction, significantly slowing the ring's movement. With *n_c_* ≥ 3, the ring becomes easily entangled in the thread, halting its motion entirely. Compared to the high pre‐stress induced by curling, the small pre‐stress resulting from bending a straight ribbon into a ring has a negligible impact on locomotion velocity.

The rotary‐to‐linear motion conversion can be simply described using screw theory, which gives a linear relationship between the angular velocity *ω* and the linear motion speed *v* as

(1) v=ηωp

where *η* is the conversion efficiency (with *η* = 1 representing no slippage) and *p* is the screw pitch. The angular velocity *ω* of the twisted ring depends on the temperature gradient across the ring induced by the photothermal irradiation and the ring geometry,^[^ ^32^ ^]^ characterized by the ring radius *r*, ribbon width *w*, and twist number *N*. For a ring exposed to perpendicular IR light at a distance *d*, *ω* scales as ^[^ ^32^ ^]^ (see more details in Supporting Information),

(2) $$\omega \propto N\kappa \alpha /[\arcsin h\left(Nw\kappa \right)\;d^{2}]$$

where *α* is the thermal expansion coefficient of the LCE materials. This indicates that *ω* increases as *r*, *w*, and *d* decreases, and as *N* increases. Thus, a higher linear speed is expected for a twisted ring with a smaller radius, more twists, and a thinner ribbon under a shorter irradiation distance. This aligns with experimental results shown in Figure 1E,F. Figure 1E demonstrates that *ω* increases slightly from ≈0.36 to ≈0.40 rad s^−1^ as *N* increases from 6 to 12 at *d* = 10 cm and *w* = 3 mm. The missing data in Figure 1E for the ring with *d* = 6 cm is due to the supercoiling when overheated at some extreme scenarios^[^ ^32^ ^]^ (e.g., *N* = 12 and *d* = 6 cm) and thus loses its functionality. Unlike the distinct free motions observed in the Möbius^[^ ^35^ ^]^ or Seifert^[^ ^37^ ^]^ ribbon‐based LCE ring actuators with even or odd twist numbers, the parity of twists in the ring does not affect its flipping angular velocity and thus the linear motion speed. This is because self‐motion of the hanging twisted ring is confined to the track due to its curling around the thread. Compared to the twist number, the ribbon width and irradiation distance show more pronounced effects (Figure 1E,F). For example, as *w* increases from ≈1.2 to ≈4 mm, *ω* decreases from ≈0.62 to ≈0.34 rad s^−1^ at *d* = 10 cm and *N* = 9 (Figure 1F). Similarly, increasing *d* from ≈6 to 12 cm causes *ω* to drop from ≈0.92 to ≈0.45 rad s^−1^ at *w* = 1.2 cm and *N* = 9 (Figure 1F).

**Figure 2:**
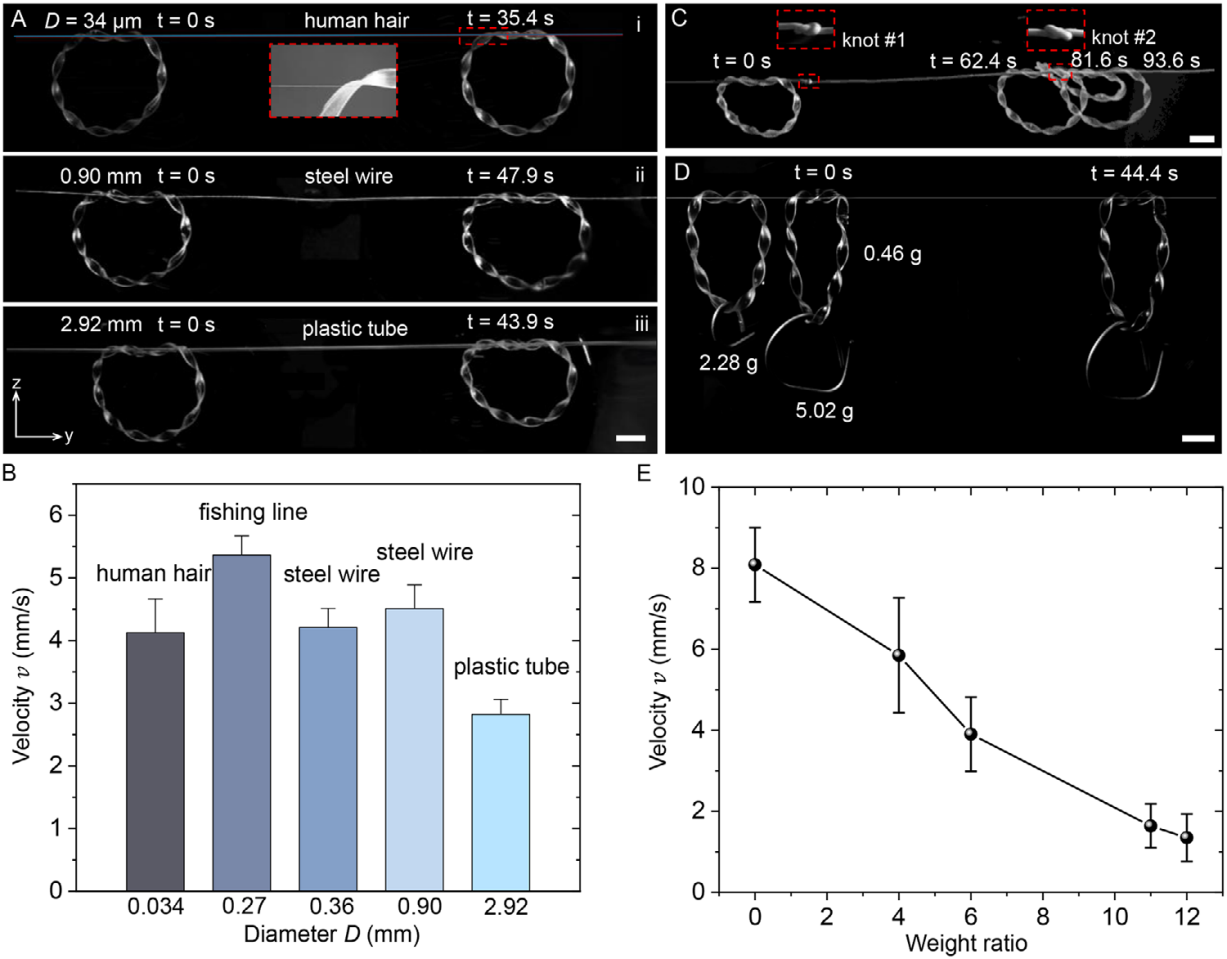
Autonomous linear motion and load transport on tracks of various materials and sizes. A) Linear motion on different‐sized tracks of a human hair (i), steel wire (ii), and plastic tube (iii). B) The measured average linear moving velocities on tracks of various materials and sizes. C) Passing through two spaced knots on a steel wire track. The inset shows the image of the knot. D) Transporting different weight loads along the track. E) Transporting velocities as a function of carrying load to self‐weight ratio. Scale bars in (A), (C), and (D): 1 cm.

A similar autonomous rotary‐to‐linear motion conversion can be achieved on straight tracks ranging from micron to millimeter scales, made of various polymer and metal materials (Movie S3, Supporting Information). **Figure** **2**A,B show that replacing the fishing line (diameter *D* = 0.27 mm) with either microscale threads, like human hair (*D* = 34 µm), or millimeter‐scale tracks, such as a plastic tube with *D* = 2.92 mm, which is close to the ribbon width (*w* = 3 mm), the twisted soft ring robot is capable of self‐moving along both the human hair (Figure 2A‐i) and large plastic tube tracks (Figure 2A‐iii) with average velocities of 4.2 mm s^−1^ and 2.8 mm s^−1^, respectively. A similar motion is observed on metal threads, such as submillimeter‐sized steel wires (Figure 2A‐ii,B). The friction tests reveal that the self‐moving linear speed *v* of the ring (Figure 2B) follows the same trend as the measured friction forces *f_friction_* between the LCE material and tracks made of different materials (Figure S9, Supporting Information). It shows that *v* decreases as *f_friction_* increases, with the ring achieving the highest speed on the fishing line due to its minimum frictional force and the slowest speed on the plastic tube due to its maximum friction force (Figure 2B; Figure S9, Supporting Information).

The flexibility of the rotating twisted ring allows it to self‐adapt to tracks with abrupt diameter changes, such as knots, enabling it to autonomously navigate obstacles without getting stuck. Figure 2C shows a steel wire thread with two knots spaced along the track, each with the same size but mirrored orientations with opposite‐level ends. Knot #1 has a lower left end, while knot #2 has a higher left end, creating higher resistance. As the soft ring encounters and passes through these knots, it adapts by rotating its curling soft body around the obstacles, successfully navigating both the lower and higher ends (Movie S4, Supporting Information).

Furthermore, the soft ring is capable of autonomously carrying and transporting loads along the track (Movie S5, Supporting Information). When carrying loads at its bottom, the soft ring, hanging on a fishing line (*D* = 0.27 mm), deforms into an oval shape due to the load's gravity (Figure 2D). Despite this deformation and the resulting shortened contact lines with the thread, the soft ring continues to self‐move along the track, transporting the object autonomously. However, the transport speed decreases dramatically as the load weight increases (Figure 2E). For example, the speed drops from 8 mm s^−1^ (without load) to 1.35 mm s^−1^ when carrying a load of 5.52 g, which is 12 times the ring's own weight, causing the ring to deform into an inverted triangular shape under the heavy load (Figure 2D). Further increase in load weight results in greater resistance, making transportation more difficult.

### 2.2 Autonomous Navigation in Two‐Dimensional (2D) Space

Next, we explore the autonomous navigation of the twisted ring in 2D space under constant photothermal actuation, including climbing slopes and moving along plane curves of various shapes.

**Figure 3:**
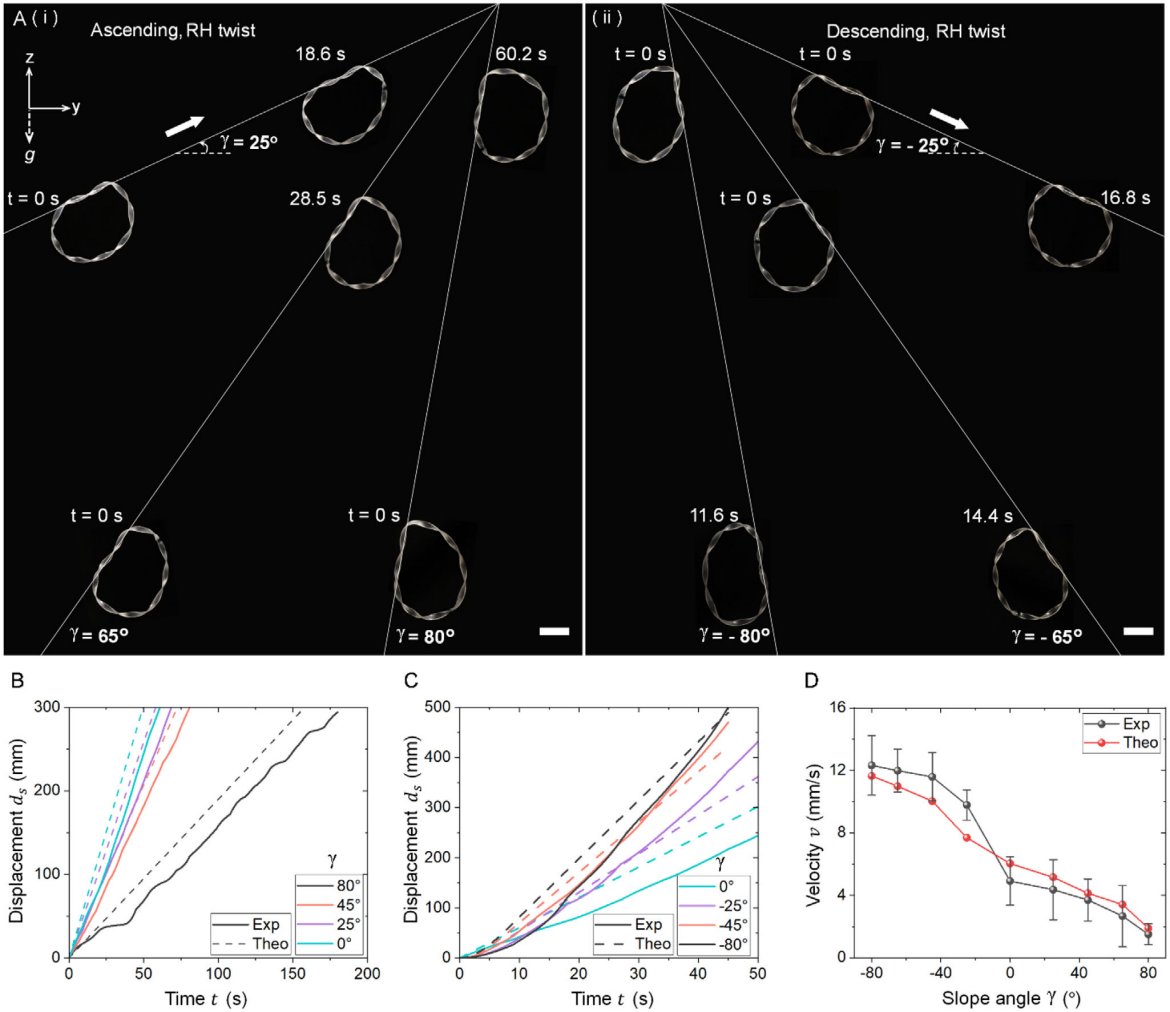
Autonomous ascending and descending sloped tracks. A) A right‐handed twisted ring robot ascending and descending various sloped tracks with slope angle *γ* increasing from 25° to 80° (i) and decreasing from −25° to −80° (ii), respectively. The ring has a ribbon width 2 mm and 9 twists. Scale bar: 1 cm. B,C) Comparison between theory and experiments on the ascending and descending displacement versus time for different sloped tracks. D) Comparison between theory and experiments on the ascending and descending velocities versus track's slope angles.

**Figure** **3**A shows a front‐view setup of sloped tracks (*y*‐*z* plane) with mirrored positive and negative slope angles *γ*. Gravity acts downward along the negative *z*‐axis. When exposed to constant IR light along the positive *x*‐axis (outward direction), a right‐handed twisted ring can autonomously climb tracks with varying positive slope angles, overcome gravity, and can even ascend slopes as steep as 80° (Movie S6, Supporting Information). When the slope angle becomes negative, the same twisted ring autonomously moves downward on slopes with angles of up to −80°. Similar to the bi‐directional motion along horizontal tracks, reversing either the twisting chirality or the light's incident direction switches its ascending or descending directions. Measurements of movement along both positive and negative sloped tracks indicate an approximately linear relationship between travel displacement and time (Figure 3B,C), reflecting a nearly constant linear speed. As expected, the descending speed is significantly faster than the ascending speed due to gravity. Figure 3D shows that as *γ* decreases from 0° to −80°, the speed increases dramatically from ≈4.8 to ≈12.5 mm s^−1^, accelerated by the gravitational component along the track (*mg* sin*γ*, where *m* is the mass of the ring and *g* is the acceleration due to gravity). Conversely, as *γ* increases from 0° to 80°, the speed drops sharply from ≈4.8 to ≈1.7 mm s^−1^ as the ring overcomes the gravitational force *mg* sin*γ* while ascending. The remarkable ability to autonomously ascend and descend steep tracks with angles of up to 80° is attributed to the anchoring effect of the twisted ring curled around the thread, highlighting the advantage of track‐guided soft robots for challenging tasks like vertical climbing or descending. Compared to the theoretical predictions of linear motion speed derived from the measured angular velocity via screw theory, the measured descending speed is slightly higher and the ascending speed is slightly lower. This discrepancy is attributed to gravity‐induced slippage during both ascending and descending, as shown by the small perturbations in the displacement‐time curves in Figure 3B,C, while the screw theory assumes no slippage. Slippage accelerates the ring's descent but slows its ascent. In addition to the gravity, the friction force also plays an important in ascending and descending slopes. As *γ* increases from −80° (descending) to 80° (ascending), the initial rotating speed of the ring decreases dramatically from ≈0.55 to ≈0.08 rad s^−1^ (Figure S10, Supporting Information). For the same twisted ring on the thread, the friction forces always align approximately with the ring's moving direction. Thus, during climbing, the friction forces need to overcome the gravity and thus slow down its linear speed, while for descending, the gravity component aligns with the friction force for speeding up.

**Figure 4:**
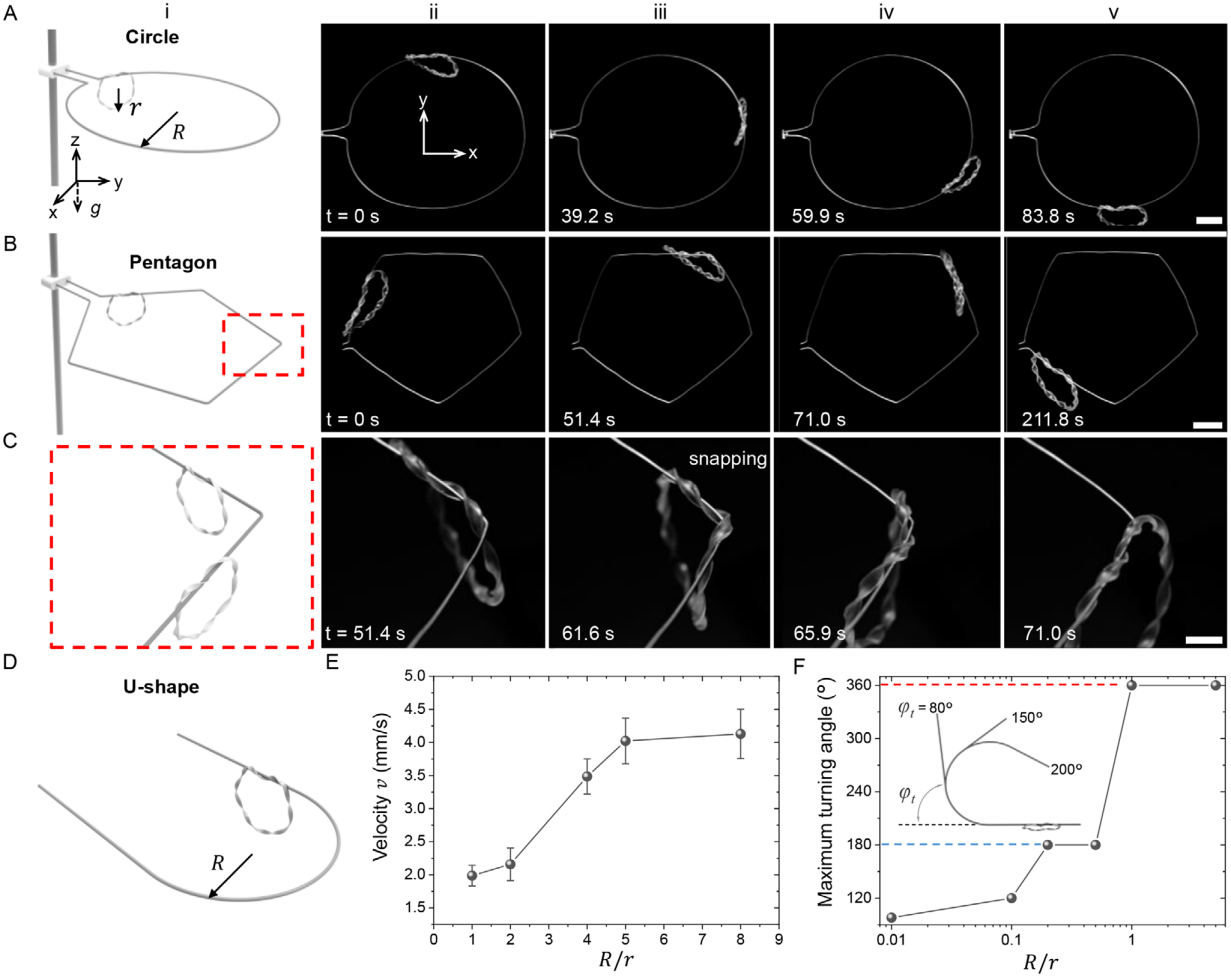
Autonomous navigation along plane curves. A) Navigation along a circular steel wire track with the schematic of set up in (i). B) Navigation along a pentagon‐shaped steel wire track with the schematic of set up in (i). C) Zoom‐in images in (B) showing passing through the vertex via snapping. D) Schematic of a U‐shaped track with a turning radius of *R*. E) Average velocity as a function of the ratio of turning radius *R* to ring radius *r* in the U‐shaped track with a relatively larger turning radius (*R*/*r* ≥ 1). F) Maximum turning angle as a function of *R*/*r* for tracks with relatively smaller turning radius (*R*/*r* ≤ 1). Scale bars in (A,B): 2 cm and in (C): 1 cm.

**Figure** **4**A,B show the autonomous navigation of the ring robot along horizontal planar tracks in three representative geometric shapes, including circular, pentagonal, and U shapes under constant photothermal actuation (with an array of fixed IR emitters positioned 5 cm away from the track and steel wires used as tracks, see Experimental Section for details). Unlike linear tracks, navigating a circular track requires continuous directional adjustment due to the curvature. Figure 4A demonstrates the soft ring robot (radius *r* = 2 cm) successfully converting rotary motion into autonomous orbiting along a circular track (radius *R* = 6.5 cm, *R*/*r* = 3.25) (Movie S7, Supporting Information), adapting to the curvature through interactions between the twisted soft body and the track, which is also well captured by the corresponding FEA simulation (Movie S7, Supporting Information). Navigating polygonal tracks, such as a pentagon, presents additional challenges due to the abrupt directional changes and small turning radius at each vertex (Figure 4B,C), resulting in a large energy barrier. Figure 4B shows that the twisted ring robot successfully navigates the pentagon‐shaped track's turning points by leveraging spontaneous local snapping to overcome the energy barrier (Figure 4C; Movie S8, Supporting Information). At each vertex, the soft ring untwists locally, storing elastic energy (Figure 4C‐iii), which is then rapidly released to snap past the turning point and facilitate navigation.

To evaluate the soft ring robot's adaptability to plane curves with different curvatures and sharp angle changes, a U‐shaped track with an adjustable turning radius *R* is designed (Figure 4D). The U‐shaped track features a turning angle of *φ_t_* = 180°. As expected, a smaller turning radius demands a higher actuation force, making self‐turning more difficult. Experimental results show that for the U‐shaped track, as the ratio of turning radius *R* to ring radius *r*, i.e., *R*/*r*, decreases from 5 to 1, the speed reduces from 4 to 2 mm s^−1^ (Figure 4E). Figure 4F summarizes the maximum turning angle *φ_t‐max_* as a function of *R*/*r*, where *R*/*r* represents the minimum turning radius for self‐navigation through a given angle. At *R*/*r* = 1, *φ_t‐max_* = 360°, indicating that the ring can autonomously navigate a complete circle. As *R*/*r* decreases to 0.2, the ring robot can still navigate the small‐radius U‐shape track, with *φ_t‐max_* dropping to 180° (Figure 4F). Further reductions in *R*/*r* lead to a progressive decrease in *φ_t‐max_*. At an extremely small turning radius of *R*/*r* = 0.01, *φ_t‐max_* decreases to 100°, corresponding to the lower limit for a transition angle of 80° in a polygonal track.

### 2.3 Autonomous Navigation in 3D Space

**Figure 5:**
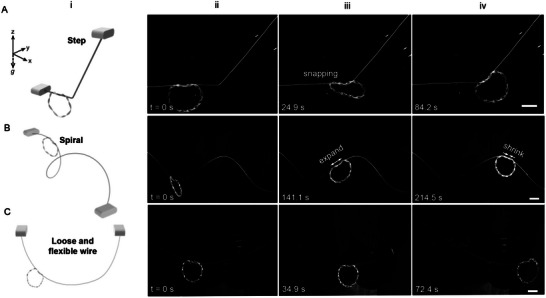
Autonomous navigation along stationary and dynamically shape‐shifting space curves. A) Navigation along a spatial step‐like steel wire track with the schematic of set up in (i). B) Navigation along a spiral steel wire track with the schematic of set up in (i). (B) Navigation along a loose, flexible, dynamically changing track of free‐hanging thin fishing line with the schematic of set up in (i). Scale bars in (A‐C): 2 cm.

Furthermore, we explore the twisted soft ring robot's autonomous navigation capabilities in more challenging 3D space along both stationary and dynamically changing space curves under constant photothermal actuation (see Materials and Methods for details). Gravity acts downward along the negative *z*‐axis. **Figure** **5**A‐i shows a simple step‐like space curve made of steel wire, composed of a horizontal thread and an inclined thread with an inclined angle of 50°, where the two threads are offset along the *x*‐axis and not in the same *y‐z* plane. The soft ring robot successfully transits from the horizontal to the inclined track via untwisting‐induced snapping (Figure 5A‐iii), and then autonomously climbs the slope (Figure 5A; Movie S9, Supporting Information).

We demonstrate the robot's autonomous navigation along a more complex 3D spiral‐like rigid track, which features varying radii of curvature and torsion (Figure 5B‐i). The soft ring robot self‐adapts, descending along the spiral despite the changing curvatures and torsions. As it moves through a wave trough, the robot autonomously adjusts its body posture by tilting its ring plane to accommodate the curvature changes, then climbs up a twisted curve (Figure 5B‐ii‐iv; Movie S10, Supporting Information). Notably, to ascend the spiral, the curling distance between the ring and the thread spontaneously elongates, increasing the interaction area to provide greater propulsion and overcome gravity (Figure 5B‐iii). After reaching the wave crest and descending, the curling distance autonomously shortens (≈10%) due to the assistance of gravity (Figure 5B‐iv). This is also captured by the corresponding FEA simulation result (Movie S10, Supporting Information).

Lastly, beyond rigid, fixed space curved tracks, we explore the robot's adaptability to a more challenging, flexible, and loose 3D curved track with real‐time dynamically shifting shapes and curvatures. The 3D concave‐shaped track is a self‐hanging polymer thread (fishing line) with its two ends at the same height along the *z*‐axis but offset along the *x*‐axis (Figure 5C‐i). Unlike rigid tracks, the loose, flexible track deforms under the weight of the soft ring (Figure 5C‐ii). As the ring moves, its gravitational force acts as a dynamically moving load, spatiotemporally changing the track's shape both locally and globally, creating a unique challenge for track‐guided soft robots. We demonstrate that the robot's rotary‐to‐linear motion conversion also works on this loose, flexible track, as it dynamically adapts to changes in the track shape, descending and ascending the curve autonomously (Figure 5C‐ii–iv; Movie S11, Supporting Information).

## 3 Discussion

We demonstrated an aerial tram‐like soft LCE twisted ring robot capable of autonomously navigating along pre‐defined tracks in 3D space under constant photothermal actuation. Despite its compliant soft body, the ring robot exhibits strong autonomous load‐carrying and transport capabilities. The free‐hanging soft ring robot self‐flips around its circular centerline, driven by the IR light‐induced temperature gradient. Curling the soft hanging ring to the track, combined with its twisted structure, facilitates converting the self‐rotary motion into autonomous linear movement along the track via screw theory. This autonomous rotary‐to‐linear motion conversion can be applied to a variety of tracks, including those made of different polymers and metals, spanning micro‐to‐millimeter scales, steep inclines, and even knotted obstacle tracks. The mechanism is effective for both stationary and dynamically changing tracks in diverse 2D and 3D geometries. These include planar tracks that demand continuous directional adjustments, such as in circular paths, sharp transitions with small turning radii in non‐circular tracks, 3D tracks with varying curvatures and torsions, and dynamically changing tracks whose shape and curvature shift in real time due to the robot's movement. The robot's strong self‐adaptability to diverse tracks is enabled by adaptive, local interactions between the twisted soft ring body and the track, such as snapping to overcome energy barriers at abrupt track transitions and intelligently adjusting its curling distance for ascending and descending 3D curves.

Compared to steering motion guided by light^[^ ^34^, ^35^, ^38^, ^39^, ^40^ ^]^ or magnetic fields^[^ ^11^, ^12^, ^21^ ^]^ and autonomous free movement^[^ ^22^, ^23^, ^24^, ^25^, ^26^, ^27^, ^28^, ^29^, ^30^, ^31^ ^]^ in soft mobile robots, the aerial track‐guided autonomous motion under constant photothermal actuation offers several advantages (see more details in Supporting Information). First, it transcends the 2D plane limitations seen in most soft robots,^[^ ^1^, ^2^, ^3^ ^]^ significantly expanding operational capabilities into 3D space. In principle, the aerial tram‐like soft robot can navigate between any two locations in 3D environments by connecting them with multi‐path tracks. The flexibility of these multi‐path aerial tracks enhances the robot's ability to navigate in more challenging, unstructured 3D environments with obstacles.

Second, predefined tracks allow for precise control of complex, multi‐dimensional motion paths by confining movement along the track,^[^ ^32^, ^33^ ^]^ which is difficult for free‐moving soft robots^[^ ^11^, ^12^, ^21^, ^22^, ^23^, ^24^, ^25^, ^26^, ^27^, ^28^, ^29^, ^30^, ^31^, ^34^, ^35^, ^38^, ^39^, ^40^ ^]^ due to their flexible, highly deformable bodies.

Third, constant photothermal actuation, combined with aerial tracks, enables autonomous navigation in multidimensional space and simplifies motion control, overcoming the complexity of stimuli‐steered 2D/3D motion that requires intricate spatiotemporal control of light ^[^ ^34^, ^35^, ^38^, ^39^, ^40^ ^]^or magnetic fields.^[^ ^11^, ^12^, ^21^ ^]^ We note that the patterned and fixed IR lights are used to simulate a constant light working environment. It eliminates the complicated spatiotemporal control of light sources with the mobile soft robots to drive their spatial motion in 3D environments. Despite its added complicity in the settings, it simplifies control to achieve complicated motions under constant light.

We envision that the track‐guided autonomous soft robots made of photothermally responsive materials such as liquid crystal polymers and hydrogels,^[^ ^34^, ^35^, ^38^, ^39^, ^40^ ^]^ under constant actuation, will offer transformative potential for precision navigation and transport in complex 3D environments, under a constant energy‐absorbing environment. This makes them ideal for applications in confined or unstructured environments, such as medical devices, soft adaptive transport and automation systems, and space exploration.

## 4 Experimental Section

### Fabrication of Twisted LCE Ring

The twisted LCE rings were fabricated by gluing the two ends of a twisted LCE ribbon that was manufactured by following the two‐stage reaction method in the previous work.^[^ ^32^ ^]^ Two g of 1,4‐Bis‐[4‐(3‐acryloyloxypropyloxy) benzoyloxy]‐2‐methylbenzene (RM257) (Wilshire Company, 95%) was fully dissolved into 0.7 g of toluene (Sigma–Aldrich, 99.8%) at 85 °C under stirring. Then, 0.012 g of (2‐hydroxyethoxy)‐2‐methylpropiophenone (HHMP; Sigma–Aldrich, 98%), 0.42 g of 2,2′‐(ethylenedioxy) diethanethiol (EDDET, Sigma–Aldrich, 95%), and 0.19 g of pentaerythritol tetrakis (3‐mercaptopropionate) (PETMP, Sigma–Aldrich, 95%) were then mixed and added to the solution. The solution was stirred at 85 °C for 20 min. After cooling down to room temperature, 0.006 g of catalyst, dipropylamine (DPA; Sigma–Aldrich, 98%) was then added and stirred at room temperature for 3 min. The solution was poured in the mold and let stand overnight for a fully reaction. The sample is left in the mold and dried at 75 °C for 24 h. The twisted LCE ribbon was obtained by first stretching the dried ribbon to ≈150% of its initial length and then twisted and exposed under UV irradiation for 10 min to finish the crosslinker process. The two ends of the twisted ribbon were bonded using UV epoxy resin (Limino) for 3 min under 395 nm UV flashlight to achieve the twisted LCE ring. The two ends were glued using UV adhesive resin with compatible geometrical connections to preserve the twisting chirality.

### Twisted Ring Navigating Tracks

The IR emitters (150 W, Diameter: 108 mm) were used as the photothermal actuation sources. To obtain a constant IR radiation field, emitters were 50 mm spaced. Two to four emitters were mounted and patterned in series on retorted stands, depending on the size of required photothermal area and the tracks. The fishing line (Berkley Trilene XL Monofilament) was used for most of the testing demonstrations without further modification unless otherwise stated. The steel wires (AAWG: 19 and 27, OD: ≈0.9 and ≈0.36 mm) were purchased from Master Wire Supply. The plastic pipe was purchased from McMaster‐Carr (OD: ≈3 mm). The steel wires were shaped into desired geometry (circular, pentagon, U‐shape, and spiral), and their ends were fixed to the 3D printed holders. The shapes of the track with a certain radius were obtained by encircling the steel wire on a 3D printed cylinder with the desired radius.

### Characterization Methods

The infrared videos and images were taken by an infrared camera (FLIR A655sc). The radiation of the IR emitter was obtained by a radiation sensor (TD‐8553, PASCO Scientific). The friction test was conducted by dragging the wires of multiple materials (see Figure S9, Supporting Information) in between two LCE sheets (size: 30 mm × 20 mm). The pressed normal force (4 N) onto the LCE sheets was controlled by a screw linear stage. The friction‐drag distance curves were measured and recorded by Instron 5944.

### Finite Element Analysis (FEA) Simulation

The Finite Element Analysis (FEA) simulation was performed using the commercial software Abaqus/Explicit to model autonomous linear, circular, and spiral motions of the LCE ring. The ring, which had ten twists, was modeled with a radius of 25 mm, width of 2.5 mm, and thickness of 0.6 mm. The LCE material was assumed to be linear elastic, with Young's modulus of 100 MPa, Poisson's ratio of 0.38, and density of 10^3^ kg m^−3^. The wire in the simulation was modeled as a fixed rigid body, with hard contact interaction between the ring and wire surfaces, characterized by a friction coefficient of 0.3. The simulation of linear motion was conducted in a multistep process. In the first step, the twisted ring was placed on a short, curved wire (hidden in the video), which initially passed through the ring. This short wire was connected to a straight one with a length of 250 mm and a radius of 0.5 mm. A displacement boundary condition was applied to drag the ring onto the straight wire. In the second step, viscous pressure loads with a magnitude of 10^−10^ and gravity were applied to bring the system to a static equilibrium. In the third step, an actuation moment of 0.01 N mm was applied at eight equally spaced positions along the ring to simulate its self‐flipping motion. The simulations for circular and spiral motion followed a similar approach to the linear motion simulation. After dragging the ring from the short wire onto the circular or spiral wire, viscous damping was applied to bring the system to a static state. The actuation moments were then loaded to flip the ring forward. For the circular wire, the radius was 100 mm. In the case of the spiral wire, the radius was 40 mm, and the pitch was 200 mm.

## Conflict of Interest

The authors declare no conflict of interest.

## Author Contributions

F.Q. and J.Y. proposed the idea. F.Q. designed and performed the experiments. C.Z. conducted a finite element analysis simulation. H.Q. and H.S. characterized motion. F.Q., C.Z., and J. Y. wrote the paper. J.Y. supervised the research. All the authors contributed to the discussion, data analysis, and editing of the manuscript.

## Supporting information



Supporting Information

Supplemental Movie 1

Supplemental Movie 2

Supplemental Movie 3

Supplemental Movie 4

Supplemental Movie 5

Supplemental Movie 6

Supplemental Movie 7

Supplemental Movie 8

Supplemental Movie 9

Supplemental Movie 10

Supplemental Movie 11

## Data Availability

The data that support the findings of this study are available from the corresponding author upon reasonable request.
