# Supplementary material for: Aerial Track‐Guided Autonomous Soft Ring Robot
Source: Adv Sci (Weinh). 2025 Apr 25;12(26):2503288. doi: 10.1002/advs.202503288 (PMC12245120; doi:10.1002/advs.202503288)
Supplement: Supplementary file 1 — Supporting Information [file ADVS-12-2503288-s012.pdf]

ADVANCED SCIENCE
Open Access

Supporting Information

for *Adv. Sci.*, DOI 10.1002/advs.202503288

Aerial Track-Guided Autonomous Soft Ring Robot

*Fangjie Qi, Caizhi Zhou, Haitao Qing, Haoze Sun and Jie Yin**

Supporting Information

Aerial track-guided autonomous soft ring robot

Fangjie Qi[a], Caizhi Zhou[a], Haitao Qing[a], Haoze Sun[a], Jie Yin[a,1]

[a] Department of Mechanical and Aerospace Engineering, North Carolina State University, Raleigh, NC 27695, USA

[1] Corresponding author: E-mail: jyin8@ncsu.edu

This supplementary information includes:

SI text

Table S1

Figure S1-S6

Captions for supplementary movies (Movie S1-S11)

References

Supplementary text

Discussion on the effect of ring geometries on flipping velocity

By creating the temperature field, a temperature gradient on both sides of the ring is the reason why the ring has flipping motions. We describe the relationship between the flipping velocity, ω_f and twisted ring's geometries, twist number, N, curvature of the ring, κ_s, thickness of the ribbon, t, ribbon width, W_S, and ribbon length, L to derive the following[1, 2]:

$$\omega_f \propto \frac{N\kappa_s\alpha\Delta TL}{\operatorname{arcsinh}(NW_S\kappa_s)} \tag{S1}$$

Where α represents the thermal expansion coefficient. Also, supposing the intensity of the IR emitter remains constant, by inverse square law (See supplementary **Fig. S8**):

$$I = \frac{P}{4\pi d^2} \tag{S2}$$

Where P is the strength of the IR source, the radiation energy of the emitter decreases as the square of the distance, d, from the source of the point of detection.

Also, Stefan-Boltzmann law states that

$$I = e\sigma A(T^4 - T_C^4) \tag{S3}$$

where e, σ, A represents emissivity, Stefan-Boltzmann constant, and radiating area. Easy to see

$$\Delta T = T_C - T_{ambient} \propto \frac{1}{d^2} \tag{S4}$$

Combining Eq. S1 and Eq. S4, we derive:

$$\omega_f \propto \frac{N\kappa_s\alpha L}{\operatorname{arcsinh}(NW_S\kappa_s)d^2} \tag{S5}$$

Comparison with our previous work [1]

In [1], we demonstrated periodic flipping, spinning, and orbiting of a soft robot driven by bonding defects in a twisted ring topology. That work explored autonomous motion on hot plates or in confined spaces with walls. Compared to our previous work, the current work represents significant advancements, not only in terms of distinct and intriguing autonomous spatial navigation and transport, enabled by the dramatically different aerial tracks and the rotary-to-linear motion conversion mechanism, but also in the broad field of autonomous mobile soft robots with precise path control using aerial tracks. These innovations ensure that the novelty and significant of this work remain unchallenged. Below, we outline the key distinctions:

First, despite the similar twisted ring topology, our previous paper reports a periodic self- orbiting soft ground robot operating on hot plates, while the current work introduces an aerial tram-like autonomous soft robot navigating multidimensional aerial tracks under constant remote photothermal actuation. These aerial tracks overcome the limitations of planar ground motion in previously studied land robots, enabling autonomous navigation and transport in 3D space.

Second, the mechanisms driving autonomous motion in these two works are fundamentally different. In the self-orbiting soft robot on hot plates, motion is driven by defect-induced symmetry breaking in the force balance. In contrast, the aerial tram-like soft robot in this work uses a rotatory-to-linear motion conversion, facilitated by adaptive interactions between the twisted ring and the tracks, for autonomous, track-guided movement. While the defect plays a crucial role in enabling periodic self-orbiting motion in the previous study, it has no role in this work.

Table S1. A comparison of other LCE actuators and this work.

References	[3]	[4]	[5]	[6]	[7]	[8]	[9]	[10]	[11]	[12]	This work
Locomotion	Linear strain	Crawlin g	Linear strain	Crawlin g	Linear strain	Shape morphin g	Bending	Shape morphin g	Shape morphin g	Shape morphin g	Track following
Material	LCE hollow fiber	LCE-liquid metal	LCE-heatin g thread	LCE	CL-LCE	LCE	LCN	LCE	DE-LCE	MXen e-LCE	LCE
Actuation	Therm al	Induct ion	IR	Heate d surfac e	UV	Heate d surfac e	Visibl e light	UV	UV	Therm al	IR
Fastest linear speed	10 s/cycle	0.6 BL/mi n	4 % /s	0.25 BL /min	2.5 % /s	Not discus sed	20 s /cycle	Not discus sed	20 % /min	180 s/cycl e	8 BL/min
Carry load while moving	×	×	×	×	×	×	×	×	×	×	√
Adapt to environment change	Not discuss ed	Not discuss ed	Not discuss ed	×	Not discuss ed	×	×	×	×	×	√
Maximum climbing angle	Not relevan t	Not discuss ed	Not relevan t	Not discuss ed	Not relevan t	Not discuss ed	Not relevan t	Not relevan t	Not discuss ed	Not discuss ed	85°
Controllable moving trajectory	×	√	×	×	×	×	×	×	×	×	√
Adjustability to multiple materials	Not relevan t	√	Not relevan t	×	Not relevan t	×	×	×	×	×	√
Universal working environment	×	√	√	√	×	√	×	×	×	×	√

Compared to recent work published in LCE-based actuators and soft robots, our work distinct itself in terms of locomotion mode, fast linear speed, object transportation, adaptability to environment changes, slope climbing, and controllable moving trajectory etc as listed in Table S1. (1) Different from the crawling and shape morphing on substrates, our work shows the unprecedented capability of autonomously following and navigating aerial tracks under constant lights. (2) It also shows fast linear speed of 8 body length

(BL)/minute, which is over 10 times faster than the speed of 0.2 BL/min in Ref. [4]. (3) It shows the unique capability of transporting objects through autonomous motion by carrying external load. (4) It shows the unprecedented adaptability to changing environments, including adapting to dynamically changing tracks and handling challenging paths with obstacles. (5) It shows strong ability for slope climbing, ascending and descending steep tracks with slopes over 80°. (6) The track-guided motion provides a simple way to control its spatial motion along the tracks, which is challenging to be achieved in previous studies.

Supplementary figures

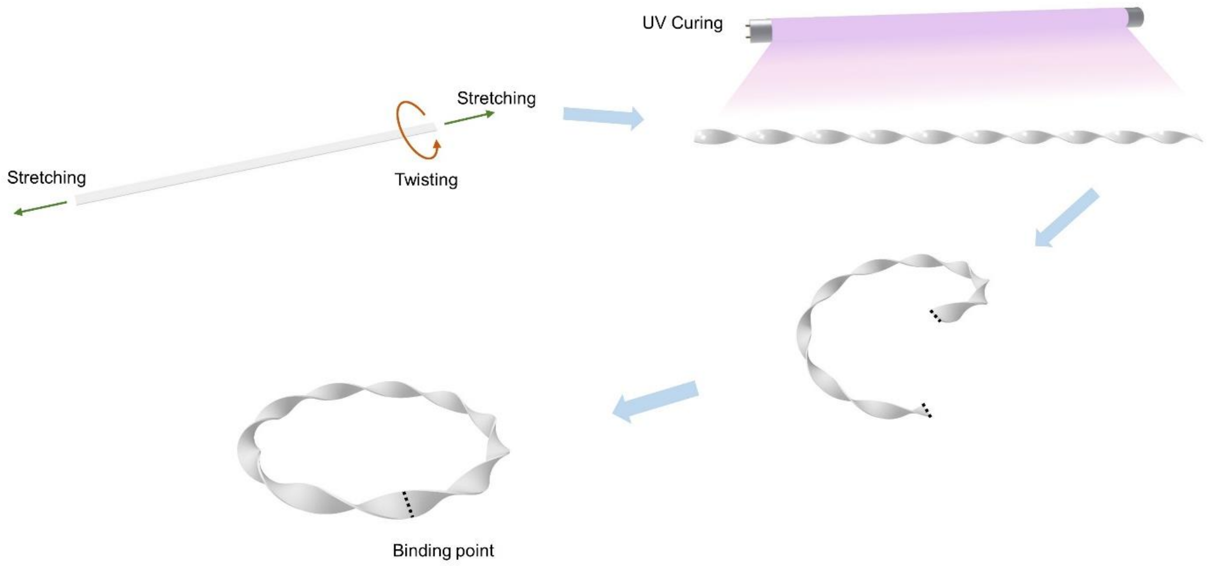

Figure S1. The schematic of fabrication process of the soft ring robot.

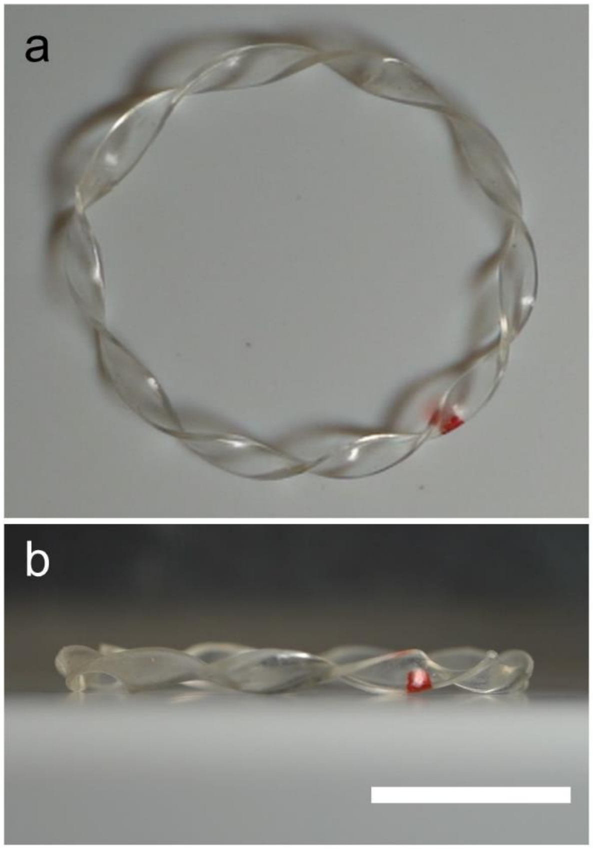

Figure S2. The photograph of the twisted LCE ring. The top view (a), and side view (b) of the soft ring robot. (Scale bar: 2 cm) The red marker shows the binding position from a twisted ribbon to a twisted ring.

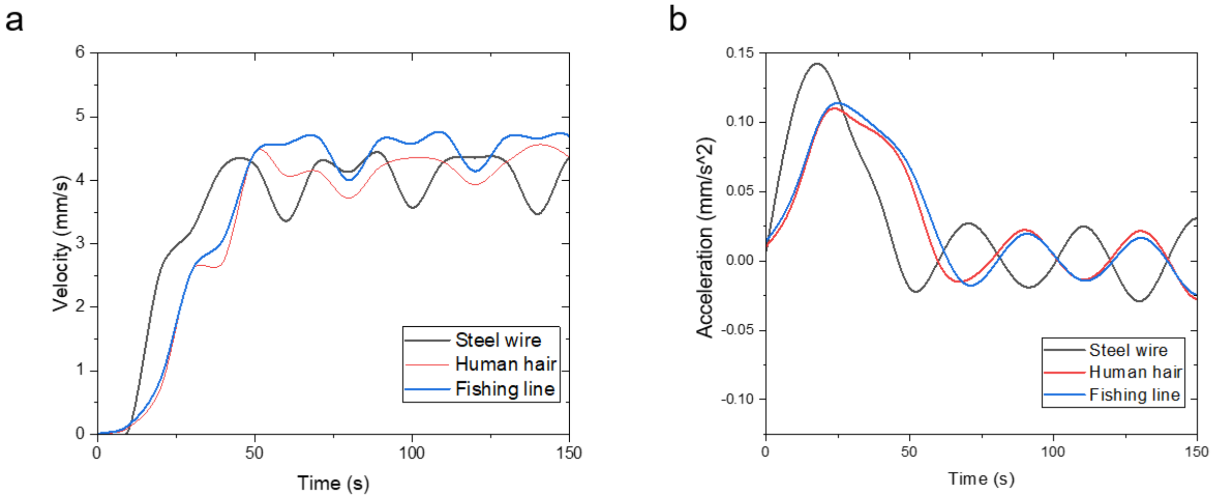

Figure S3. **Linear velocity and acceleration of the binding site.** (a) Linear velocity of the binding point. During the initial (accelerating) phase, the driving torque must overcome the friction torque to initialize the ring. The ring stabilizes after the acceleration, but still oscillates because of the binding point, which does not provide driving torque but consumes extra energy, thus a small deacceleration and acceleration can be observed periodically. (b) Acceleration of the binding point over time.

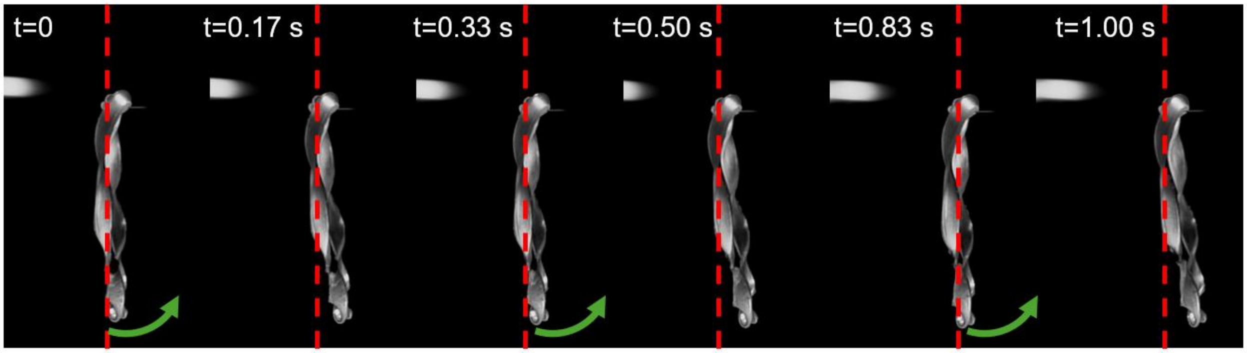

Figure S4. **Rolling friction between the track and the LCE ring.** The red dashed line represents the vertical line. The rolling friction torque is balanced by a torque generated by gravity. A tiny oscillation can be observed.

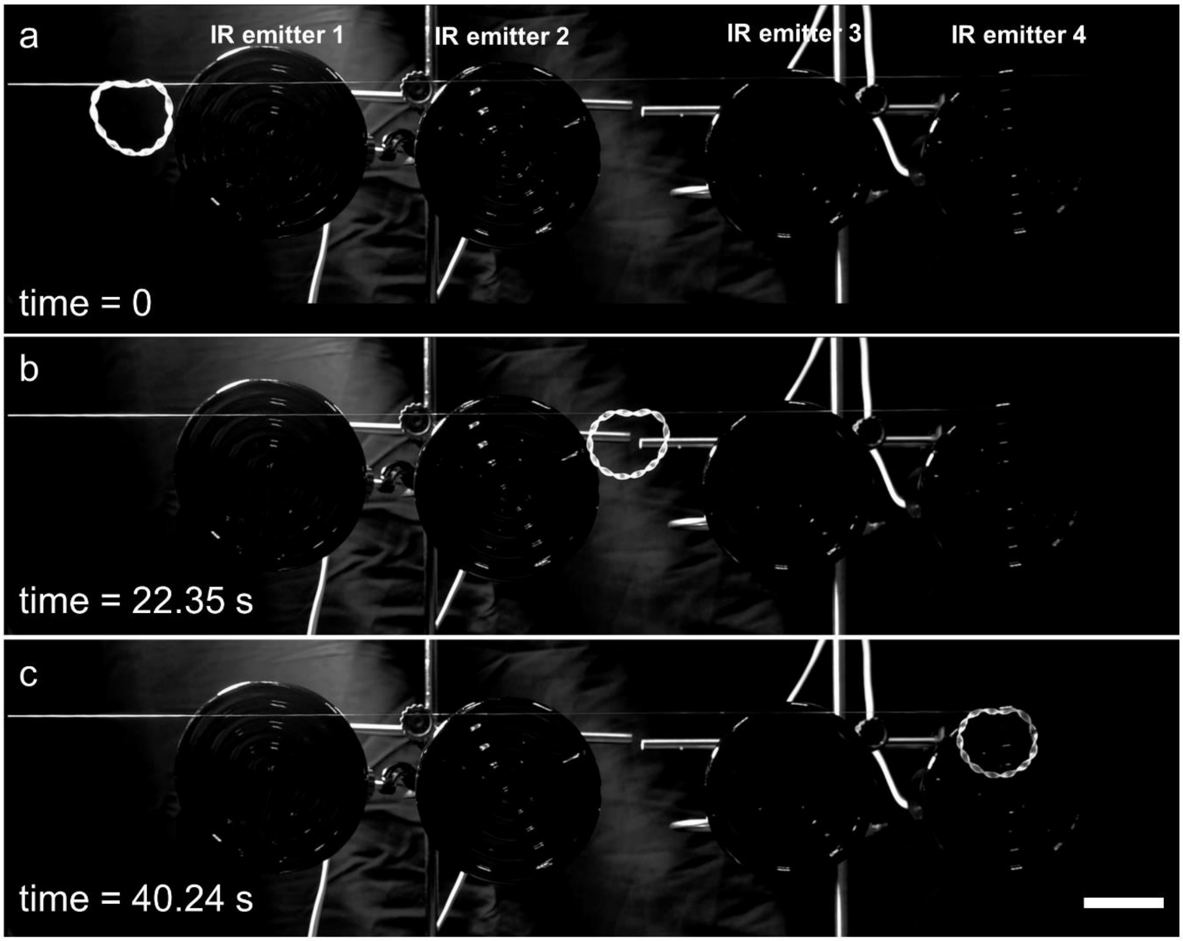

Figure S5. **The photographs of a soft ring robot climbing on a line before an IR array**. The array simulates a constant and parallelled photothermic field. The ring robot ($w_s = 4\,mm, \theta_s = 30°$, right-handed twists) starts from initial position (a), after preheating stage then reaches at position in (b), and finally ends at position in (c). The IR emitters are not necessarily close to each other as the photothermic field is scattered and the ring can still travel for a certain distance even if it is off from the center of the emitter (Scale bar: 5 cm)

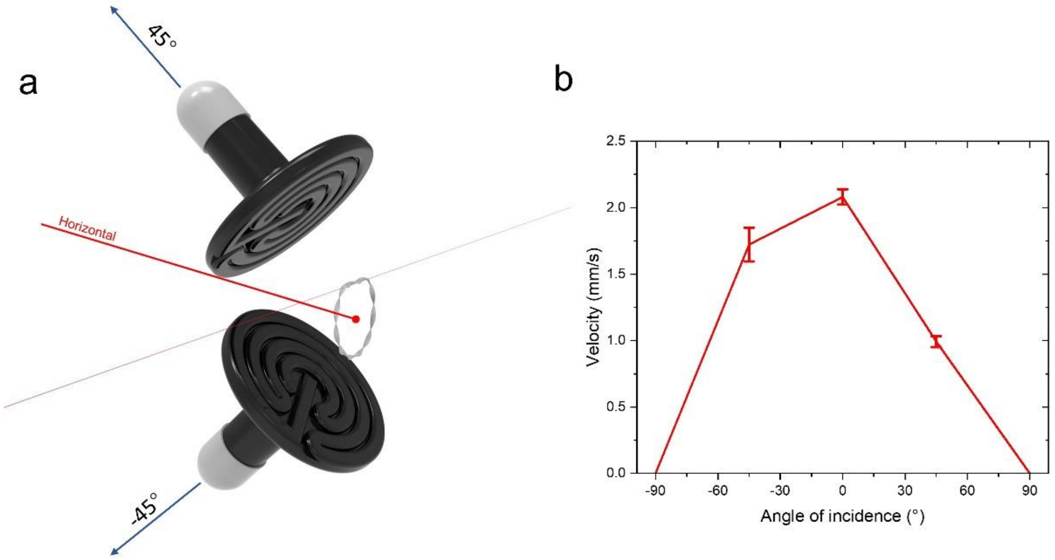

Figure S6. **Linear velocity as a function of angle of incidence**. (a) Schematic of experimental setup to show how linear velocity changes as a function of angles of incidence for the emitter. (b) Experimental results of (a). The velocity drops to zero when the angle is close to -90° (the emitter is placed vertically below the robot) or 90° (the emitter is placed vertically above the robot).

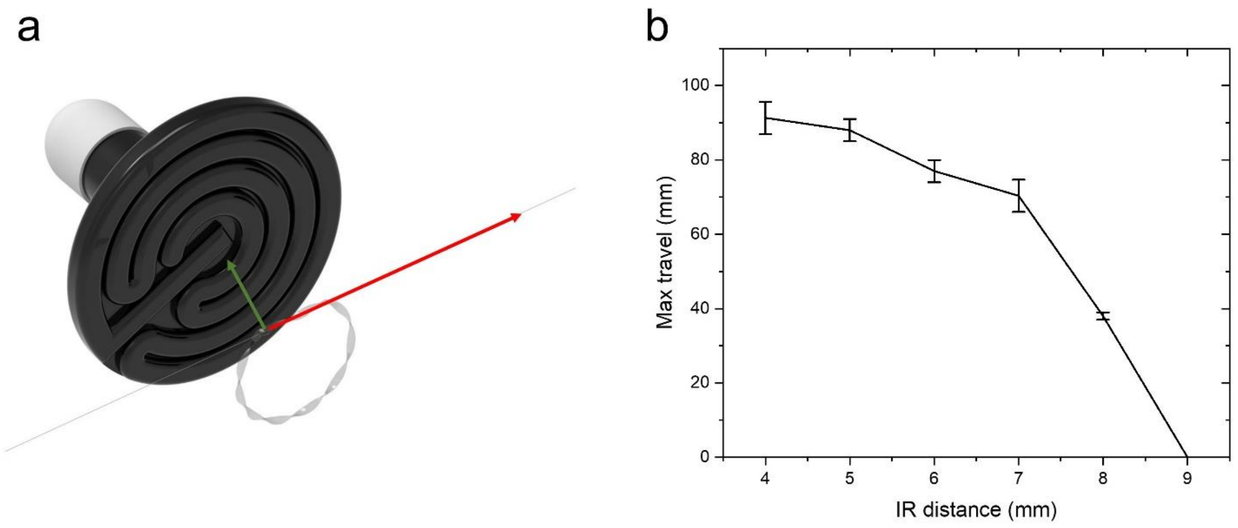

Figure S7. **The maximum travelling distance from the center of the emitter as a function of IR distance**. (a) Schematic of a soft ring robot's locomotion under IR photothermic field. (b) Experimental results of the maximum travelling distance (red arrow) of the robot ($w_s = 4\ mm, \theta_s = 30°$, right-handed twists), starting from the center of the parallelled IR emitter (Power = 150 Watts), as a function of the distance between them (green arrow).

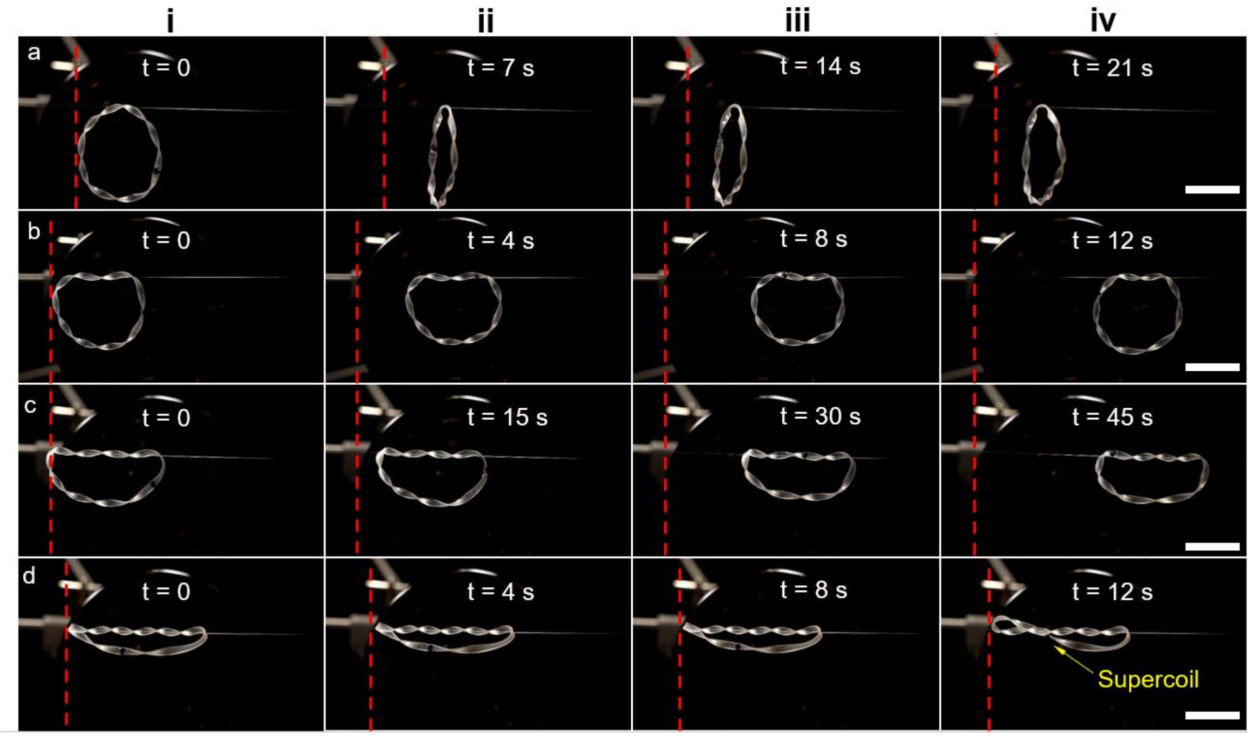

Figure S8. **Photographs of twisted LCE ring's travelling distance on track influenced by the number of curls**. As wrapping rounds n_c changes from 0 (a), 1(b), 2(c) and 3(d), the corresponding linear motion and travelling distance indicate the optimized n_c = 2. The red dashed lines mark the initial positions of the ring. (Scale bar: 2 cm)

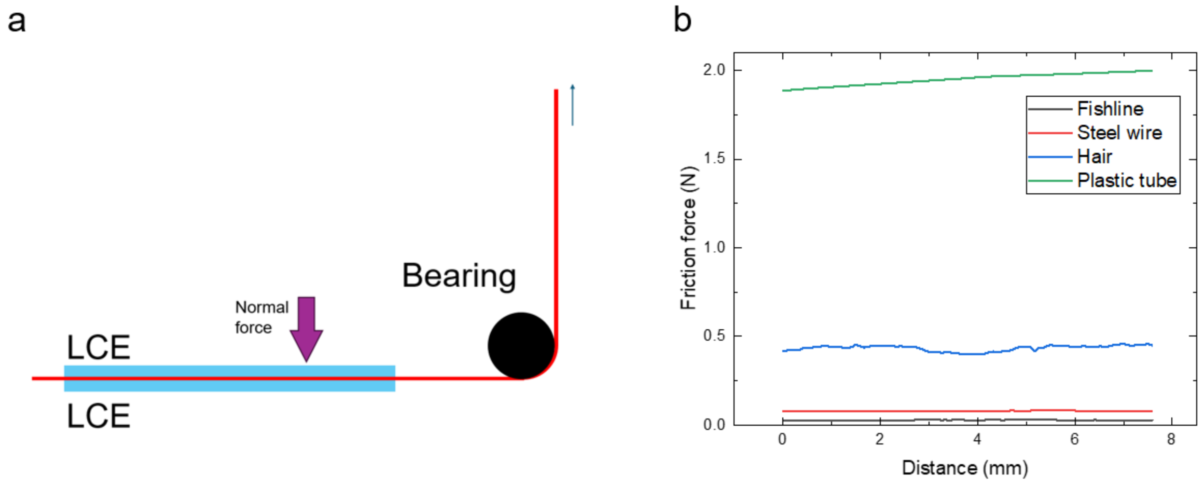

Figure S9. **Friction test between LCE and wires of multiple materials**. (a) Schematic of the setup of the friction test (The size of the LCE sheet is 4 mm × 3 mm. The normal force is kept at 4 N for all experiments). (b) Sliding friction force as a function of pulling distance of the wire after the friction stabilizes.

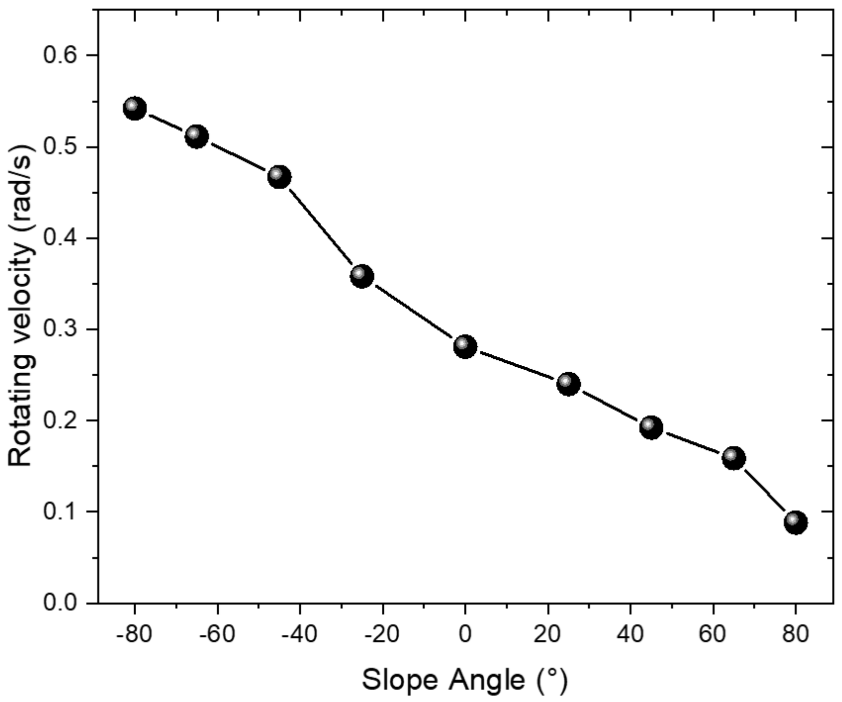

Figure S10. **Initial rotating velocity as a function of slope angle.**

Supplementary movie captions

Movie S1. The twisted LCE ring autonomously crawls on a line. The moving directions of the LCE ring is dependent on both the chirality of the twists, as well as the directions of the photothermic field.

Movie S2. Finite element analysis (FEA) simulation of the twisted LCE ring moving on the line.

Movie S3. The twisted LCE ring crawls on tracks of various materials and outer diameters.

Movie S4. The twisted LCE ring overcomes knots on the line.

Movie S5. The comparison of the twisted LCE ring's locomotion on the line with different load carrying (~5 and ~10 times of the ring's weight).

Movie S6. The twisted LCE ring crawls on sloping lines. The inclined angles vary from 25° to 80°.

Movie S7. The twisted LCE ring travels across a track of a circular shape and FEA simulation.

Movie S8. The twisted LCE ring traverses a track of a pentagon.

Movie S9. The process of the twisted LCE ring passing through an inclined angle on the track.

Movie S10. The twisted LCE ring travels across a spiral shaped wire and FEA simulation.

Movie S11. The twisted LCE ring travels across a loose wire.

References

1. Qi, F.J., et al., *Defected twisted ring topology for autonomous periodic flip-spin-orbit soft robot.* Proceedings of the National Academy of Sciences of the United States of America, 2024. **121**(3).
2. Baumann, A., et al., *Motorizing fibres with geometric zero-energy modes.* Nature Materials, 2018. **17**(6): p. 523.
3. Ma, J.Z., et al., *Liquid Crystal Elastomer Hollow Fibers as Artificial Muscles with Large and Rapid Actuation Enabled by Thermal-Pneumatic Enhanced Effect.* Advanced Functional Materials, 2024. **34**(37).
4. Maurin, V., et al., *Liquid Crystal Elastomer-Liquid Metal Composite: Ultrafast, Untethered, and Programmable Actuation by Induction Heating.* Advanced Materials, 2024. **36**(34).
5. Chen, W.H., et al., *Knotted Artificial Muscles for Bio-Mimetic Actuation under Deepwater.* Advanced Materials, 2024. **36**(27).
6. Escobar, M.C. and T.J. White, *Fast and Slow-Twitch Actuation via Twisted Liquid Crystal Elastomer Fibers.* Advanced Materials, 2024. **36**(34).
7. Fan, Q.Y., et al., *Cluster-Triggered Self-Luminescence, Rapid Self-Healing, and Adaptive Reprogramming Liquid Crystal Elastomers Enabled by Dynamic Imine Bond.* Advanced Materials, 2024. **36**(31).

8. Herman, J.A., et al., *Digital Light Process 3D Printing of Magnetically Aligned Liquid Crystalline Elastomer Free-forms.* Advanced Materials, 2024. **36**(52).
9. Li, Z.Z., et al., *Reconfigurable Visible Light-Driven Liquid Crystalline Network Showing Off-Equilibrium Motions Enabled by Mesogen-Grafted Donor-Acceptor Stenhouse Adducts.* Advanced Materials, 2024. **36**(49).
10. Yue, L.S., et al., *Vacuum Thermoforming of Optically Switchable Liquid Crystalline Elastomer Spherical Actuators.* Advanced Materials, 2024. **36**(30).
11. Zhang, C.C., et al., *Repeatedly Programmable Liquid Crystal Dielectric Elastomer with Multimodal Actuation.* Advanced Materials, 2024.
12. Yang, Y.W., et al., *Near-Infrared Light-Driven MXene/Liquid Crystal Elastomer Bimorph Membranes for Closed-Loop Controlled Self-Sensing Bionic Robots.* Advanced Science, 2024. **11**(2).
